# A Rare Instance of Concordant Charcot-Marie-Tooth Disease and Familial Partial Lipodystrophy Type 2

**DOI:** 10.7759/cureus.109361

**Published:** 2026-05-21

**Authors:** Mark A Bachir, Iyawnna Hazzard, Alexander S Bachir, Gavin Chima, Biljinder Chima

**Affiliations:** 1 Medicine, California Northstate University College of Medicine, Elk Grove, USA; 2 Research, California Northstate University College of Medicine, Elk Grove, USA; 3 Medicine, California Health Sciences University (CHSU), Clovis, USA; 4 Family Medicine, Jesuit High School, Arden-Arcade, USA; 5 Family Medicine and Sports Medicine, Rocklin Family Practice and Sports Medicine, Rocklin, USA

**Keywords:** axonal polyneuropathy, cardiac surveillance, case report, charcot-marie-tooth disease, cmt2, familial partial lipodystrophy, fpld2, laminopathy, lmna variant, peripheral neuropathy

## Abstract

Lamin A/C (LMNA)-related laminopathies can involve adipose tissue, peripheral nerves, skeletal muscle, and the heart. We report a 65-year-old woman with a seven-year history of progressive distal sensorimotor neuropathy, burning pain in both feet, loss of hand dexterity, and worsening steppage gait. Physical examination demonstrated distal intrinsic hand and foot atrophy, absent reflexes, distal sensory loss, pes cavus, claw and hammer toe deformities, hindfoot varus, limb lipoatrophy, and relative cervicofacial/central adiposity. Electromyography and nerve conduction studies (EMG/NCS) showed a length-dependent, axonal-predominant mixed sensorimotor polyneuropathy consistent with a hereditary neuropathy and Charcot-Marie-Tooth (CMT)-like phenotype. Genetic testing identified a heterozygous pathogenic lamin A/C variant consistent with familial partial lipodystrophy type 2 (FPLD2); exact variant nomenclature was not available in the records accessible to the authors. Available metabolic data showed impaired fasting glucose, with glucose at 109 mg/dL and hemoglobin A1c (HbA1c) at 5.6%, while triglycerides and low-density lipoprotein (LDL) cholesterol were controlled at 139 mg/dL and 54 mg/dL, respectively. Fasting insulin, C-peptide, and homeostatic model assessment for insulin resistance (HOMA-IR) were not available; therefore, the available data support dysglycemia and increased metabolic risk rather than definitive biochemical confirmation of severe insulin resistance. Management included custom orthopedic footwear with supramalleolar orthotic (SMO) inserts, endurance-focused physical therapy, gabapentin for neuropathic pain, metformin for dysglycemia, atorvastatin for lipid and atherosclerotic cardiovascular disease (ASCVD) risk reduction, antihypertensive therapy for blood-pressure control, and longitudinal cardiac surveillance with electrocardiogram (ECG), echocardiography, and ambulatory rhythm monitoring. This case highlights the multisystem nature of LMNA-related disease and the importance of recognizing overlapping adipose, neurologic, metabolic, and cardiac manifestations as part of a unified disease process requiring coordinated neuromuscular, endocrine, and cardiology follow-up.

## Introduction

Charcot-Marie-Tooth (CMT) disease is an autosomal dominant inherited condition that causes damage to peripheral nerves, leading to various neuropathic symptoms, including numbness, tingling, pain, muscle weakness, and atrophy [1], and is estimated to affect approximately 700,000 to 2 million people worldwide [1]. Prolonged untreated neuropathy in CMT can also lead to foot deformities, such as pes cavus and hammer toes [1]. The most common types of CMT include Charcot-Marie-Tooth type 1 (CMT1) and Charcot-Marie-Tooth type 2 (CMT2), which affect the myelin sheath and axon of the peripheral nerves, respectively, leading to peripheral nerve problems [1].

Familial Partial Lipodystrophy (FPLD) is another autosomal dominant, inherited disease where adipose tissue is unevenly distributed around an individual’s body [2,3]. FPLD is genetically classified into six subtypes, with Familial Partial Lipodystrophy type 2 (FPLD2), due to lamin A/C (LMNA) mutations, being the most common form [2,3]. The estimated prevalence of FPLD is between 0.3 and 4.7 cases per million [3-5]. Individuals with this condition have marked loss of adipose tissue in their extremities, along with excessive adipose deposition in their facial tissue, neck, and liver [2,3]. This uneven fat distribution may lead to severe conditions, including diabetes, pancreatitis, heart disease, or hypertension [3].

LMNA is a nuclear envelope protein that helps maintain nuclear structure and regulates cellular signaling and gene expression [2-4]. Pathogenic variants in LMNA can cause laminopathies, a group of multisystem disorders that may involve adipose tissue, peripheral nerves, skeletal muscle, and cardiac tissue [2,3]. In peripheral neuropathy, demyelinating disease primarily affects the myelin sheath surrounding nerves, whereas axonal disease primarily affects the nerve fiber itself. Pes cavus refers to a high-arched foot deformity and is a common clinical clue to hereditary neuropathy such as CMT disease [1]. In this case presentation, we describe a rare case of a 65-year-old female with the coexistence of CMT and FPLD.

## Case presentation

**Figure 1:**
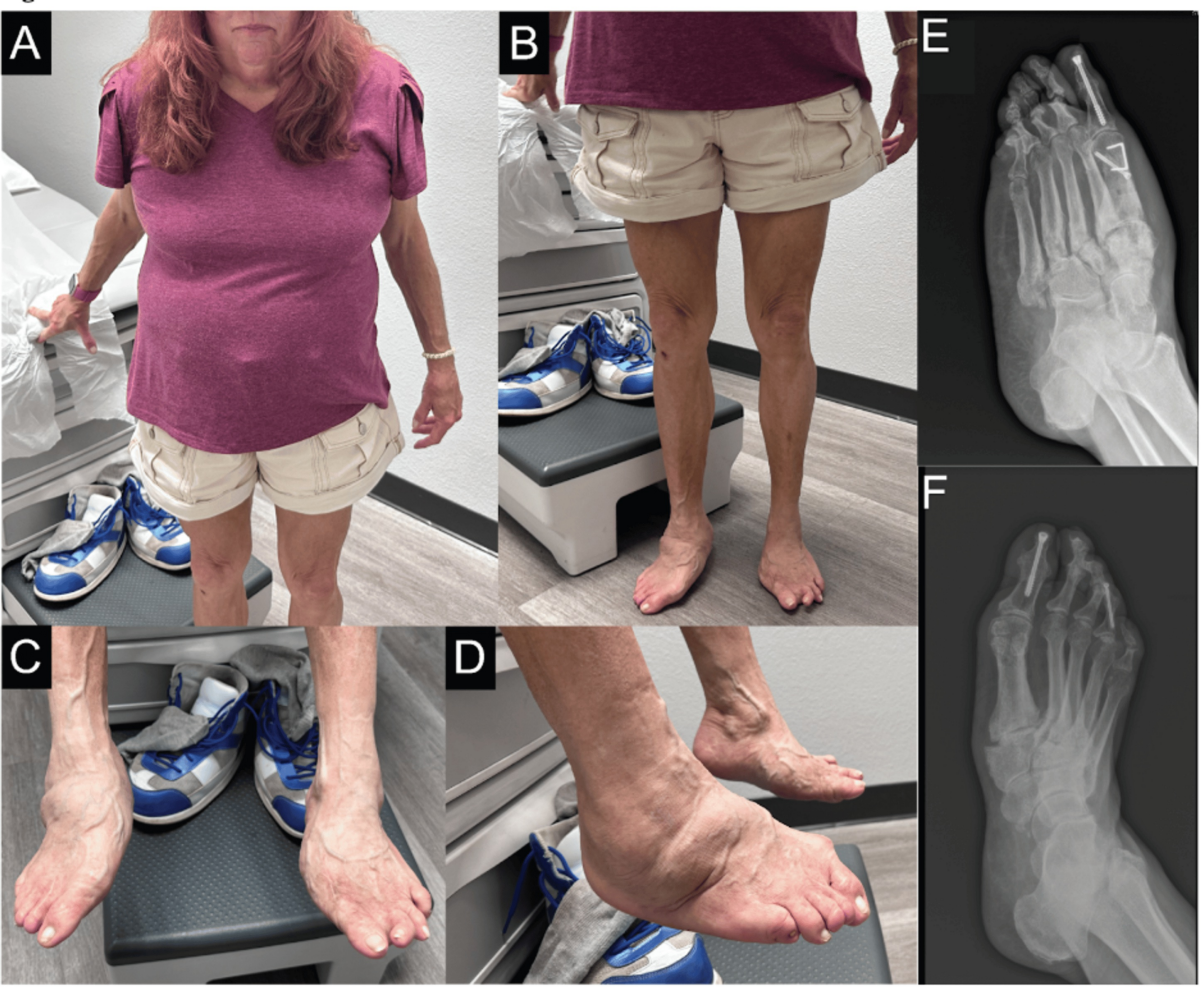
Deidentified clinical and radiographic features of familial partial lipodystrophy and Charcot-Marie-Tooth type foot deformity Photo of patient demonstrating prominent adipose distribution to (A) face and abdomen with (B) atrophy to upper and lower extremities consistent with Familial Partial Lipodystrophy and Charcot-Marie-Tooth foot deformity in frontal (C) and lateral (D) views and non-weight-bearing, oblique X-rays of (E) left and (F) right feet illustrating Charcot-Marie-Tooth deformity.

We present a case of a 65-year-old female with a seven-year history of progressive distal sensorimotor neuropathy and limb lipoatrophy who was followed in a multidisciplinary neuromuscular program from 2022 to 2024. Beginning in 2017, she reported burning pain and numbness in her feet bilaterally with a gradual loss of hand dexterity and a progressively worsening steppage gait. She also had decreased subcutaneous fat over the extremities with relative facial/neck adiposity, consistent with FPLD, as well as bilateral pes cavus, claw and hammer toes, and hindfoot varus foot deformities consistent with CMT disease (Figure 1). She ambulates independently in custom high-ankle orthopedic shoes with supramalleolar orthoses (SMO) inserts and remains independent in her activities of daily living. Family history is notable for a granddaughter diagnosed with CMT in childhood and a son with pes cavus foot deformities consistent with CMT.

During her initial multidisciplinary visit in October 2022, she described distal pain, weakness, and limited endurance. Physical examination revealed distal intrinsic hand and foot atrophy, absent reflexes, impaired pinprick sensation below the knees with relatively preserved position sense, predominantly distal weakness to her upper and lower extremities bilaterally, mild steppage gait, and no cranial neuropathy or myotonia.

Subsequent electrodiagnostic testing, including nerve conduction studies, showed absent lower-extremity sensory responses and markedly reduced distal compound muscle action potentials (CMAPs) in the legs, with prolonged distal latencies and slowed conduction. Upper extremity sensory studies showed a right-sided ulnar conduction block across the elbow in the setting of prior cubital tunnel disease, but were otherwise comparatively preserved. Needle electromyography (EMG) demonstrated chronic neurogenic changes in the upper and lower extremities, with distal muscle groups worse than proximal muscles, without a myopathic pattern. These findings were consistent with a length-dependent, axonal-dominant, mixed sensorimotor polyneuropathy most consistent with a hereditary neuropathy. Germline testing identified a heterozygous pathogenic LMNA variant associated with FPLD2. Exact variant nomenclature was not available in the records accessible to the authors.

Later surveillance laboratory evaluation on September 8, 2025, demonstrated impaired fasting glucose (glucose 109 mg/dL) and a hemoglobin A1c (HbA1c) of 5.6%. Lipid testing showed total cholesterol 139 mg/dL, triglycerides 139 mg/dL, high-density lipoprotein (HDL) 57 mg/dL, low-density lipoprotein (LDL) 54 mg/dL, and very-low-density lipoprotein (VLDL) 28 mg/dL. Creatine kinase was mildly elevated at 252 U/L, and estimated glomerular filtration rate (eGFR) was mildly decreased at 72 mL/min/1.73 m² (Table 1). The mildly elevated creatine kinase and mildly reduced estimated glomerular filtration rate were noted as nonspecific abnormalities and were not sufficient to establish primary myopathic involvement or LMNA-related renal disease.

**Table 1:** Laboratory data All laboratory values were taken from September 8, 2025. Abnormal values are indicated in bold. Reference values are for females when pertinent. *If lasting > 3 months H: high, L: low, BUN: blood urea nitrogen, eGFR: estimated glomerular filtration rate, AST: aspartate aminotransferase, ALT: alanine aminotransferase, HDL: high-density lipid, LDL: low-density lipid, VLDL: very low-density lipid. CKD: chronic kidney disease

Laboratory	Result	Reference Value
Hemoglobin A1c	5.6%	≥ 6.5% diabetes
Glucose	109 mg/dL (H)	70–99 mg/dL
Sodium	135 mmol/L	135–145 mmol/L
Potassium	3.6 mmol/L	3.5–5.0 mmol/L
Chloride	100 mmol/L	96–106 mmol/L
Bicarbonate	29 mmol/L	22–29 mmol/L
BUN	24 mg/dL (H)	7–20 mg/dL
Creatinine	0.88 mg/dL	~0.6–1.3 mg/dL
eGFR	72 mL/min/1.73 m^2^ (L)	≥90 normal, 60–89 mildly decreased, <60 suggests CKD*
Calcium	9.5 mg/dL	8.5–10.5 mg/dL
Albumin	3.8 g/dL	3.5–5.0 g/dL
Phosphorus	3.4 mg/dL	2.5–4.5 mg/dL
AST	27 U/L	10–40 U/L
ALT	34 U/L	7–56 U/L
Alkaline Phosphatase	82 U/L	44–147 U/L
Total Bilirubin	0.3 mg/dL	0.1–1.2 mg/dL
Creatine Kinase (CK)	252 U/L (H)	~20–180 U/L
Total Cholesterol	139 mg/dL	​​< 200 mg/dL
Triglycerides	139 mg/dL	<1 50 mg/dL
HDL	57 mg/dL	≥ 50 mg/dL
LDL	54 mg/dL	< 100 mg/dL
VLDL (calculated)	28 mg/dL	5–30 mg/dL

## Discussion

Patient management

CMT disease and FPLD are both associated with atrophy. In CMT, length-dependent axonal degradation is responsible for distal weakness with intrinsic foot and hand muscle wasting and gait imbalance. In FPLD2, selective loss of limb subcutaneous fat with myosteatosis drives paroxysmal fatigue, insulin resistance, and functional decline. Therefore, she was managed with an endurance-focused home physical therapy (PT) program. This included hip abduction bridges, abdominal strength training, sit-to-stand movements, wall slides, and lower-extremity stretches. Neuropathic pain was managed with gabapentin 600 mg three times daily. This medication is known to inhibit the presynaptic α2δ subunit of voltage-gated calcium channels to reduce neuronal hyperexcitability and central sensitization, making this an appropriate symptomatic therapy for the neuropathic pain seen in CMT-related axonal polyneuropathy.

Considering her confirmed LMNA pathogenic variant, patient education was provided to emphasize that laminopathies can involve concurrent pathology of the heart, peripheral nerves, and adipose tissue, so a coordinated, multidisciplinary follow-up schedule is essential [6]. LMNA mutations classically cause early-penetrant dilated cardiomyopathy with progressive conduction system disease and early-onset malignant atrial and ventricular arrhythmias. Importantly, clinically significant arrhythmias can occur even when left-ventricular systolic function is preserved [6-8]. Conduction delay or cardiac arrhythmias may precede overt cardiomyopathy and confer a risk of syncope and sudden cardiac death [7,8]. Because of this, she was referred for longitudinal cardiology care with baseline and annual electrocardiogram, echocardiography, and ambulatory rhythm monitoring, with a low threshold for cardiac magnetic resonance imaging (MRI) and electrophysiology evaluation. We also reviewed warning symptoms, including palpitations, presyncope, syncope, exercise intolerance, and dyspnea, and discussed the potential need for early device therapy given LMNA-specific arrhythmic risk [6,7]. We additionally discussed the association between LMNA variants and axonal CMT2 neuropathy, which explains her neuropathic pain and underscores that neurologic and cardiac manifestations can co-occur; ongoing neuromuscular follow-up for function, pain control, and fall prevention complements cardiac surveillance [9].

She also carries a phenotype consistent with FPLD2, also known as Dunnigan type FPLD, an LMNA-related adipose disorder associated with markedly increased cardiometabolic risk. Selective loss of peripheral subcutaneous fat with ectopic lipid deposition drives severe insulin resistance, hypertriglyceridemia, atherogenic dyslipidemia, and nonalcoholic fatty liver disease or steatohepatitis, with premature atherosclerotic cardiovascular disease (ASCVD) and ischemic events that can occur independent of traditional risk factors [10,11]. Accordingly, we instituted repeat metabolic surveillance, including fasting lipids, HbA1c, and/or oral glucose tolerance testing, liver enzymes ± hepatic ultrasound, blood-pressure monitoring, and weight and waist tracking (Table 1). We also counseled intensive lifestyle measures plus early pharmacologic risk reduction - statin therapy to achieve LDL and non-high-density lipoprotein (non-HDL) targets; fibrates or high-dose omega-3 for very high triglycerides to reduce pancreatitis risk; and consideration of metformin, a glucagon-like peptide-1 (GLP-1) receptor agonist, or a sodium-glucose cotransporter-2 (SGLT2) inhibitor for dysglycemia, as well as referral to endocrinology to assess candidacy for metreleptin in severe hypoleptinemic partial lipodystrophy [3,10,12]. She was placed on metformin to address insulin resistance and glycemic control, which is typical of FPLD-associated metabolic disease; atorvastatin to treat atherogenic dyslipidemia and lower ASCVD risk; amlodipine and hydralazine to improve blood-pressure control and reduce afterload, decreasing risk of heart failure; lisinopril for neurohormonal blockade and ventricular remodeling protection given LMNA-related cardiomyopathy risk; carvedilol to provide additional beta-blockade for arrhythmia suppression and cardioprotection; and spironolactone for mineralocorticoid receptor antagonism to mitigate adverse remodeling and heart-failure progression in the setting of LMNA-associated susceptibility to cardiomyopathy and malignant arrhythmias [3,6,7,10]. In summary, LMNA provides a unifying explanation for the triad of axonal CMT-type neuropathy, FPLD-driven metabolic disease, and a distinct susceptibility to arrhythmias and dilated cardiomyopathy. Proactive cardiology and endocrine follow-up is therefore critical to prevent sudden arrhythmic events and to reduce long-term ASCVD and heart-failure risk [6,7,10].

Genetic association of LMNA variants with concordant CMT and FPLD

To our knowledge, this is the first case that highlights the association between two rare diseases, CMT disease and FPLD2. There have been previous case reports demonstrating the coexistence of CMT1A and nondystrophic myotonia due to a peripheral myelin protein 22 (PMP22) duplication and sodium channel Nav1.4 alpha subunit (SCN4A) mutation [13]. In this case report, the proband had combined neurogenic and myogenic changes in the lower extremities, demyelinating and remyelinating biopsies of the sural nerve leading to atrophy of the distal lower extremities with simultaneous hypertrophy of the lower extremities, pes cavus, and areflexia [13]. Our case had a similar presentation with muscle atrophy of the distal extremities, foot deformities, areflexia, markedly reduced conduction velocities, and decreased CMAP amplitude as seen on EMG. However, our case differs in that our patient lacked proximal muscle hypertrophy of the lower extremities, as indicated in Figure 1B. Lastly, the concordance of CMT and FPLD was due to a mutation in LMNA as opposed to an SCN4A mutation and PMP22 duplication.

The similarities between these two cases underscore an underlying genetic similarity responsible for the parallelism between neurogenic and myogenic characteristics. Two other case reports have previously shown strong evidence of LMNA variants leading to diseases involving adipose, nervous, cardiac, and muscle tissue, such as CMT and FPLD [14]. In previous literature and systematic reviews, LMNA variant mutations have been strongly implicated with FPLD [10,14] as well as CMT [9]. The LMNA gene codes for an intermediate filament protein within the nuclear lamina, which is important for epigenetics and intracellular signaling [14]. Defects in this gene cause laminopathies and lipodystrophies, leading to fat deposition in the heart, pancreatic β-cell dysfunction causing insulin resistance, and elevated glucose production and reuptake [14].

This case has some limitations. First, fasting insulin, C-peptide, and homeostatic model assessment for insulin resistance were not available, limiting biochemical confirmation of severe insulin resistance despite the patient’s familial partial lipodystrophy phenotype and impaired fasting glucose. Second, the patient was followed in a multidisciplinary neuromuscular program from 2022 to 2024, while the laboratory data reported in Table 1 were obtained on September 8, 2025; therefore, the laboratory values should be interpreted as later surveillance data rather than initial diagnostic laboratory values. Finally, although the patient remained independent in activities of daily living and ambulated independently with custom orthopedic footwear, standardized functional outcome measures such as gait speed, formal fall-risk scores, or disability scales were not available.

## Conclusions

This case demonstrates a clinically important overlap of FPLD2 with a CMT-like axonal sensorimotor polyneuropathy in the setting of LMNA-related disease. Although multisystem laminopathy phenotypes are well recognized, this case emphasizes the importance of recognizing adipose, neurologic, metabolic, and cardiac manifestations as part of a unified disease process. Patients with this phenotype require coordinated neuromuscular care, cardiometabolic surveillance, fall-prevention strategies, and longitudinal cardiac monitoring for conduction disease, arrhythmias, and cardiomyopathy.
